# Sustained high serum malondialdehyde levels are associated with severity and mortality in septic patients

**DOI:** 10.1186/cc13155

**Published:** 2013-12-11

**Authors:** Leonardo Lorente, María M Martín, Pedro Abreu-González, Alberto Domínguez-Rodriguez, Lorenzo Labarta, César Díaz, Jordi Solé-Violán, José Ferreres, Judith Cabrera, Jose Carlos Igeño, Alejandro Jiménez

**Affiliations:** 1 Intensive Care Unit, Hospital Universitario de Canarias, Ofra, s/n. La Laguna, Santa Cruz de Tenerife 38320, Spain; 2 Intensive Care Unit, Hospital Universitario Nuestra Señora Candelaria, Crta Rosario s/n, Santa Cruz de Tenerife 38010, Spain; 3 Deparment of Phisiology, Faculty of Medicine, University of the La Laguna, Ofra, s/n. La Laguna, Santa Cruz de Tenerife 38320, Spain; 4 Deparment of Cardiology, Hospital Universitario de Canarias, Ofra, s/n. La Laguna, Santa Cruz de Tenerife 38320, Spain; 5 Intensive Care Unit, Hospital San Jorge de Huesca, Avenida Martínez de Velasco nº36, Huesca 22004, Spain; 6 Intensive Care Unit, Hospital Insular, Plaza Dr. Pasteur s/n, Las Palmas de Gran Canaria 35016, Spain; 7 Intensive Care Unit, Hospital Universitario, Dr. Negrín Barranco de la Ballena s/n, Las Palmas de Gran Canaria 35010, Spain; 8 Intensive Care Unit, Hospital Clínico Universitario de Valencia, Avda, Blasco Ibáñez nº17-19, Valencia 46004, Spain; 9 Research Unit, Hospital Universitario de Canarias, Ofra, s/n. La Laguna, 38320, Santa Cruz de Tenerife, Spain

## Abstract

**Introduction:**

There is a hyperoxidative state in sepsis. The objective of this study was to determine serum malondialdehyde (MDA) levels during the first week of follow up, whether such levels are associated with severity during the first week and whether non-surviving patients showed higher MDA levels than survivors during the first week.

**Methods:**

We performed an observational, prospective, multicenter study in six Spanish Intensive Care Units. Serum levels of MDA were measured in 328 patients (215 survivors and 113 non-survivors) with severe sepsis at days one, four and eight of diagnosis, and in 100 healthy controls. The primary endpoint was 30-day mortality and the secondary endpoint was six -month mortality. The association between continuous variables was carried out using Spearman’s rank correlation coefficient. Cox regression analysis was applied to determine the independent contribution of serum MDA levels on the prediction of 30-day and 6-month mortality. Hazard ratio (HR) and 95% confidence intervals (CI) were calculated as measures of the clinical impact of the predictor variables.

**Results:**

We found higher serum MDA in septic patients at day one (p < 0.001), day four (p < 0.001) and day eight (p < 0.001) of diagnosis than in healthy controls. Serum MDA was lower in surviving than non-surviving septic patients at day one (p < 0.001), day four (p < 0.001) and day eight (p < 0.001). Serum MDA levels were positively correlated with lactic acid and SOFA during the first week. Finally, serum MDA levels were associated with 30-day mortality (HR = 1.05; 95% CI = 1.02-1.09; p = 0.005) and six-month mortality (hazard ratio (HR) = 1.05; 95% CI = 1.02-1.09; p = 0.003) after controlling for lactic acid levels, acute physiology and chronic health evaluation (APACHE)-II, diabetes mellitus, bloodstream infection and chronic renal failure.

**Conclusions:**

To our knowledge, this is the largest series providing data on the oxidative state in septic patients to date. The novel finding is that high serum MDA levels sustained throughout the first week of follow up were associated with severity and mortality in septic patients.

## Introduction

**Figure 1:**
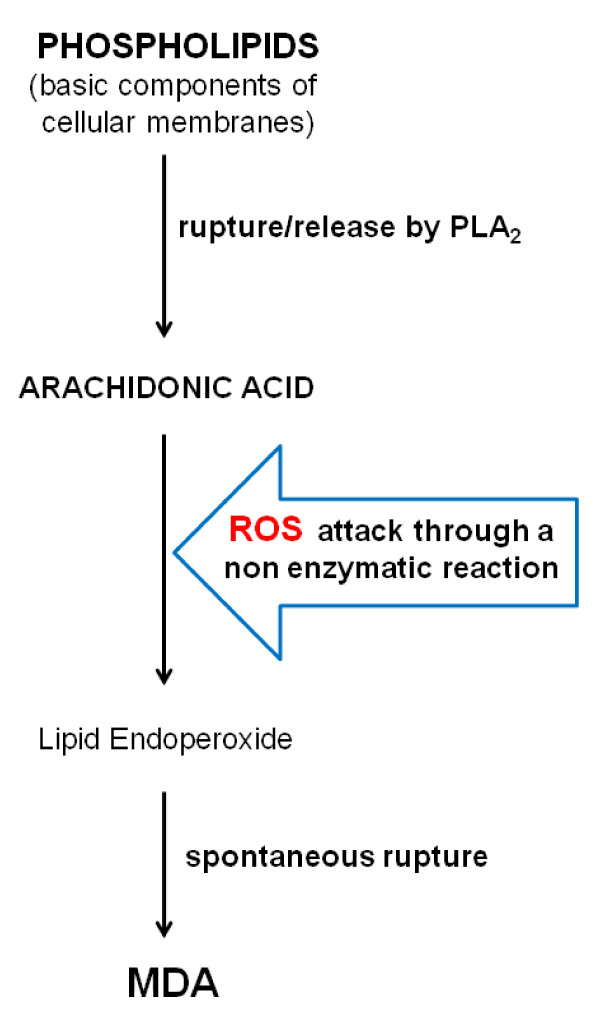
**Principal steps in the formation of MDA.** MDA, malondialdehyde; PL,phospholipase; ROS, reactive species of oxygen.

There is a hyperoxidative state in sepsis [1-5], which results from an imbalance between Doxidants and antioxidants, and includes oxidative modification of cellular macromolecules, induction of cell death by apoptosis and structural tissue damage. Malondialdehyde (MDA) is an end-product formed during oxidative stress, concretely lipid peroxidation [4,5]. It is one of several products formed during the degradation of cellular membrane phospholipids. Arachidonic acid (AA) is released due to the action of phospholipase (PL)-A_2._ Subsequently, AA is attacked by reactive species of oxygen (ROS) (principally the hydroxyl radical OH•) from mitochondria through a non-enzymatic reaction and lipid endoperoxide is formed. This lipid endoperoxide undergoes spontaneous rupture and MDA is formed in the intracellular space (Figure 1). MDA is released into extracellular space and finally into the blood. MDA has been used as an effective biomarker of lipid oxidation for more than 30 years.

Circulating MDA levels in septic patients have only been assessed in small series [6-11]. In a previous study by our group, we found higher MDA levels at diagnosis of sepsis in non-surviving than in surviving patients [12]. The objective of this study was to determine serum MDA levels during the first week of follow-up, whether such levels are associated with severity during the first week and whether non-surviving patients showed higher MDA levels than survivors during the first week.

## Methods

### Design and subjects

A multicenter, observational, prospective study was carried out in six Spanish intensive care units (ICU). The study was approved by the Institutional Review Boards of the six hospitals recruiting patients: Hospital Universitario de Canarias (La Laguna. Santa Cruz de Tenerife, Spain), Hospital Universitario Nuestra Señora de Candelaria (Santa Cruz de Tenerife, Spain), Hospital San Jorge (Huesca, Spain) and Hospital Insular (Las Palmas de Gran Canaria, Spain), Hospital Universitario Dr. Negrín (Las Palmas de Gran Canaria, Spain), Hospital Clínico Universitario de Valencia (Valencia, Spain). All patients provided written informed consent to participate in the study.

Inclusion criteria were the diagnosis of severe sepsis according to the International Sepsis Definitions Conference criteria [13]. Exclusion criteria were: age <18 years, pregnancy, lactation, human immunodeficiency virus (HIV), white blood cell count <1,000 cells/μl, solid or hematological tumor, or immunosuppressive, steroid or radiation therapy.

A total of 328 patients with severe sepsis and 100 healthy controls were included. The sample size of 100 healthy controls was arbitrary and the sample size of 328 patients was the number of patients recruited during 18 months. The controls were recruited from only one participating center, Hospital Universitario de Canarias (La Laguna, Santa Cruz de Tenerife, Spain), and were selected on the basis of their non-exposure to antioxidant agents. Community, nosocomial intra-ICU and nosocomial extra-ICU severe sepsis were included. Patients transferred from other hospitals were not included.

### Variables recorded

The following variables were recorded for each patient: sex, age, diabetes mellitus, chronic renal failure (defined as glomerular filtration rate (GFR) <60 ml/minute per 1.73 m^2^), chronic obstructive pulmonary disease (COPD), site of infection, creatinine, leukocytes, lactic acid, platelets, international normalized ratio (INR), activated partial thromboplastin time (aPTT), Acute Physiology and Chronic Health Evaluation II (APACHE-II) score [14], Sepsis-related Organ Failure Assessment (SOFA) score [15]. Severity of illness was assessed by APACHE-II and SOFA scores and lactatemia.

### Endpoints

The primary endpoint was 30-day all-cause mortality and the secondary endpoint was 6-month all-cause mortality.

### Serum MDA analysis

Blood samples were collected in citrated tubes from 328 patients with severe sepsis at days 1, 4 and 8 after diagnosis and from 100 healthy controls. Day 1 of severe sepsis was considered as the first day when the patient’s medical record contained the written diagnostic report that he/she met the consensus criteria for severe sepsis. Day 4 was considered as the day after 72 hours had elapsed, and Day 8 as the day after seven days had elapsed since the diagnostic report.

After coagulation during 20 minutes at room temperature, serum was obtained by centrifugation at 1,000 g for 15 minutes. The samples were aliquoted and frozen at −80°C until determination. The assay of MDA levels was centralized in the Department of Physiology, Faculty of Medicine (University of the La Laguna, Santa Cruz de Tenerife, Spain). Serum MDA levels were measured using the thiobarbituric acid-reactive substance (TBARS) method as described by Kikugawa *et al*. [16]. The pink complex of samples was extracted in n-butanol. Each sample was placed in a 96-well plate and read at 535 nm in a microplate spectrophotometer reader (Benchmark Plus, Bio-Rad, Hercules, CA, USA). The detection limit of this assay was 0.079 nmol/ml; the intra- and inter-assay CV were 1.82% and 4.01%, respectively. The serum concentration of MDA was expressed in nmol/ml. To avoid the possible dispersion of serum MDA level results, all the samples were processed at the same time, at the end of the recruitment process. MDA determination was performed by a laboratory technician blinded to all clinical data.

### Statistical methods

Continuous variables are reported as medians and interquartile ranges. Categorical variables are reported as frequencies and percentages. Comparisons between groups for categorical variables were carried out with chi-square test.

Comparisons of serum MDA levels among days 1, 4 and 8 in septic patients were carried out globally with Friedman and by paired groups with Wilcoxon tests.

Global comparisons of serum MDA levels separately for 30-day survivors and non-survivors using ANOVA. Comparisons between pairs of groups separately for 30-day survivors and non-survivors in MDA levels were carried out with Student’s *t*-test for repeated measures.

Comparisons of continuous variables between groups were carried out using the Mann–Whitney U test. We used the Kruskal-Wallis test to compare continuous variables in multiple groups. For these comparisons, the Bonferroni correction was applied to control for the multiple testing problem.

We performed multiple comparisons of MDA levels between control subjects and severe septic patients, and between surviving and non-surviving septic patients for each organ dysfunction, given the differences in MDA levels found by Toufekoula *et al*. according to specific organ dysfunction [10]. We also analyzed MDA levels in septic patients according to the site of infection and microorganism responsible. The criteria used to define organ dysfunction were those used in the Sepsis-related Organ Failure Assessment (SOFA) score [15]. The time point used for these analyses was Day 1 MDA levels. We only took into account each organ separately, not the association of the failure of more than one organ.

The association between continuous variables was carried out using Spearman’s rank correlation coefficient. Cox regression analysis was applied to determine the independent contribution of serum MDA levels on the prediction of 30-day and 6-month mortality. To avoid the co-linearity effect [17], we only included diabetes mellitus, bloodstream infection, chronic renal failure, lactic acid level and APACHE-II score as co-predictors. In the regression analyses we excluded age, platelet count, INR, aPTT, creatinine and SOFA score due to the co-linearity effect with the predictors included in the multiple regression using Spearman’s rank correlation test. Bivariate analysis showed no relationship between MDA levels and sex, chronic obstructive pulmonary disease, ischemic heart disease, site of infection, microorganism responsible, appropriate empiric antimicrobial treatment, so these variables were not included in the regression analyses. Hazard ratio and 95% confidence intervals were calculated as measures of the clinical impact of the predictor variables.

Receiver operating characteristic (ROC) curves were used, with MDA levels as the independent variable and 30-day mortality as the dependent variable. Thirty-day survival curves, using serum MDA level lower or higher than 4.11 nmol/mL, were represented using the Kaplan-Meier method and compared by log-rank test. A *P*-value of less than 0.05 was considered statistically significant. Statistical analyses were performed with SPSS 17.0 (SPSS Inc., Chicago, IL, USA) and NCSS 2000 (Kaysville, UT, USA).

## Results

**Figure 2:**
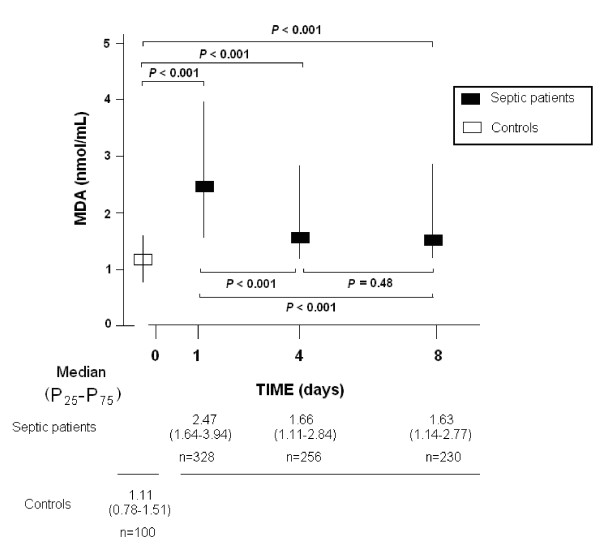
**MDA serum levels in septic patients and healthy controls.** We used Bonferroni correction to control the multiple testing problem (0.05/6 = 0.008). Thus, only *P*-values lower than 0.008 were considered statistically significant.

We found higher serum MDA levels in septic patients at Day 1 (*P* <0.001), Day 4 (*P* <0.001) and Day 8 (*P* <0.001) of diagnosis than in healthy controls, as shown in Table 1 and Figure 2.

**Table 1:** Demographic’ characteristics of healthy controls and septic patients

	**Healthy controls (n = 100)**	**Septic patients (n = 328)**	***P*-value**
Gender male - n (%)	62 (62.0)	219 (66.8)	0.40
Age - median years (p 25 to 75)	59 (47 to 70)	61 (49 to 71)	0.36
MDA - median nmol/mL (p 25 to 75)	1.11 (0.78 to 1.51)	2.47 (1.64 to 3.94)	<0.001

Comparison of demographic and clinical parameters between surviving (n = 215) and non- surviving (n = 113) septic patients is shown in Table 2. We found that non-surviving patients showed higher age, rate of diabetes mellitus, circulating levels of creatinine, lactic acid and MDA, INR, aPTT, SOFA and APACHE-II scores, and reduced platelet count, compared with surviving patients.

**Table 2:** Patients’ demographic and clinical characteristics of septic patients

	**Survival (n = 215)**	**Non-survival (n = 113)**	***P*-value**
Gender male – n (%)	146 (67.9)	73 (64.6)	0.62
Age - median years (p 25 to 75)	59 (47 to 69)	65 (56 to 74)	0.001
Diabetes mellitus - n (%)	54 (25.1)	45 (39.8)	0.008
Chronic renal failure - n (%)	13 (6.0)	14 (12.4)	0.06
COPD - n (%)	32 (14.9)	16 (14.2)	0.99
Ischemic heart disease - n (%)	24 (11.2)	12 (10.6)	0.99
Site of infection - n (%)			0.94
·Respiratory	122 (56.7)	63 (55.8)	
·Abdominal	59 (27.4)	32 (28.3)	
·Neurological	5 (2.3)	1 (0.9)	
·Urinary	12 (5.6)	6 (5.3)	
·Skin	10 (4.7)	5 (4.4)	
·Endocarditis	6 (2.8)	5 (4.4)	
·Osteomyelitis	1 (0.5)	1 (0.9)	
Microorganisms responsible - n (%)			0.89
·Unknown	114 (53.0)	61 (54.0)	
·Gram-positive	51 (23.7)	27 (23.9)	
·Gram-negative	50 (23.3)	24 (21.2)	
·Fungii	4 (1.9)	4 (3.5)	
·Anaerobe	2(0.9)	1 (0.9)	
Bloodstream infection - n (%)	29 (13.5)	18(15.9)	0.62
Empiric antimicrobial treatment adequate - n (%)			0.76
·Unknown due to negative cultures	114 (53.0)	62 (54.9)	
·Adequate	84 (39.1)	44 (38.9)	
·Unknown due to antigenuria diagnosis	4 (1.9)	3 (2.7)	
·Inadequate	13 (6.0)	4 (3.5)	
Betalactamic more aminoglycoside - n (%)	45 (20.9)	27 (23.9)	0.57
Betalactamic more quinolone - n (%)	111 (51.6)	56 (49.6)	0.73
Pa0_2_/FI0_2_ ratio - median (p 25 to 75)	183 (127 to 271)	169 (102 to 240)	0.11
Creatinine (mg/dl) - median (p 25 to 75)	1.30 (0.80 to 2.10)	1.63 (1.00 to 2.95)	0.009
Bilirubin (mg/dl) - median (p 25 to 75)	0.87 (0.50 to 1.40)	0.93 (0.50 to 2.17)	0.28
Leukocytes - median*10^3^/mm^3^ (p 25 to 75)	14.6 (9.2 to 19.4)	14.9 (6.8 to 20.4)	0.72
Lactic acid - median mmol/L (p 25 to 75)	2.00 (1.10 to 3.40)	3.55 (1.60 to 6.00)	<0.001
Platelets - median*10^3^/mm^3^ (p 25 to 75)	195 (131 to 269)	132 (67 to 224)	<0.001
INR - median (p 25 to 75)	1.25 (1.10 to 1.50)	1.42 (1.15 to 1.90)	0.002
aPTT - median seconds (p 25 to −75)	32 (28 to 39)	36 (29 to 46)	0.005
SOFA score - median (p 25 to 75)	9 (7 to 11)	11 (9 to 15)	<0.001
APACHE-II score - median (p 25 to 75)	19 (15 to 23)	23 (19 to 29)	<0.001
MDA - median nmol/mL (p 25 to 75)	2.35 (1.62 to 3.81)	3.71 (1.65 to 4.56)	<0.001

APACHE-II, Acute Physiology and Chronic Health Evaluation-II; aPTT, Activated partial thromboplastin time; COPD, chronic obstructive pulmonary disease; INR, International normalized ratio; PaO_2_/FIO_2,_ pressure of arterial oxygen/fraction of inspired oxygen; SOFA, Sepsis-related Organ Failure Assessment; data are presented as number (percentage) or median (interquartile range); Labwork was made at first day.

**Figure 3:**
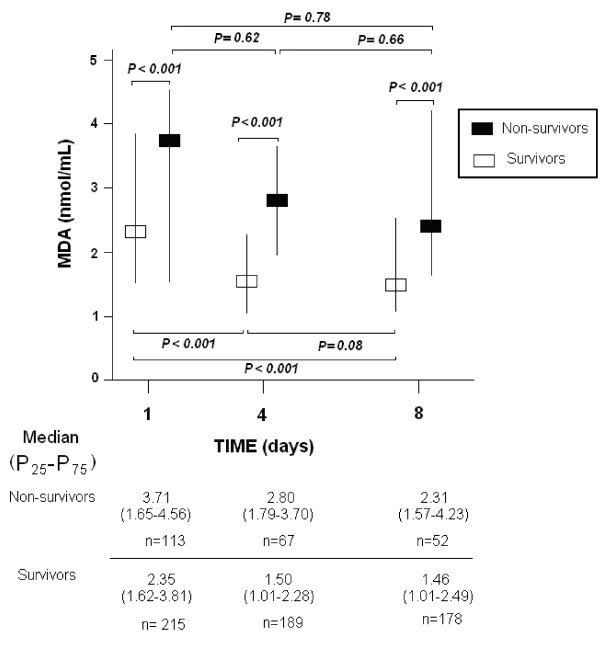
**Serum MDA serum levels in survivor and non-survivors septic patients.** We used Bonferroni correction to control the multiple testing problem (0.05/9 = 0.006). Thus, only *P*-values lower than 0.006 were considered statistically significant.

We found higher serum MDA levels in non-surviving than surviving septic patients at Day 1 (*P* <0.001), Day 4 (*P* <0.001) and Day 8 (*P* <0.001). Survivors showed a higher level of MDA at Day 1 compared with Day 4 (*P* <0.001) and Day 8 (*P* <0.001), but no difference was found between days 4 and 8 (*P* = 0.08). Non-survivors did not differ in MDA levels between Day 1 and Day 4 (*P* = 0.62) or Day 8 (*P* = 0.78) and between days 4 and 8 (*P* = 0.66) (Figure 3).

Given that some authors have found different levels of circulating MDA according to specific organ dysfunction in septic patients [10,18], we compared MDA levels in septic patients with different organ dysfunction and control subjects, and in non-surviving and surviving septic patients regardless of which organ was affected. We found higher MDA levels (*P* <0.001) in septic patients with respiratory 2.79 (1.64 to 4.31), hematologic 3.79 (2.29 to 6.37), hepatic 4.60 (2.74 to 8.04), cardiovascular 3.07 (1.77 to 4.53) or renal dysfunction 3.29 (1.96 to 5.70) than in controls subjects (1.11 (0.78 to 1.51)). We found higher MDA levels (*P* <0.001) in non-surviving than in surviving septic patients with respiratory, hematologic, hepatic, cardiovascular or renal dysfunction (Table 3).

**Table 3:** MDA serum levels in survivor and non-survivors septic patients according to each organ dysfunction

	**Survival**	**Non-survival**	***P*-value**
Respiratory	2.25 (1.50 to 3.73)	3.80 (1.97 to 6.24)	<0.001
Hematological	3.17 (2.01 to 5.01)	4.79 (3.12 to 9.71)	0.001
Hepatic	3.81 (2.15 to 6.06)	6.26 (4.18 to 10.99)	0.001
Cardiovascular	2.38 (1.56 to 3.87)	3.80 (2.06 to 6.23)	<0.001
Renal	2.61 (1.64 to 4.33)	3.90 (2.48 to 7.39)	<0.001

We used Bonferroni correction to control the multiple testing problem (0.05/5 = 0.01). Thus, only *P*-values lower than 0.01 were considered statistically significant.

There were no statistically significant differences in MDA levels in septic patients according to the site of infection (*P* = 0.06): respiratory 2.27 (1.50 to 3.83), abdominal 3.25 (1.63 to 6.03), neurological 2.36 (0.90 to 4.03), urinary 2.16 (1.76 to 4.40), skin 3.70 (1.97 to 4.56), endocarditis 3.90 (2.45 to 5.79) and osteomyelitis 4.01 (0.57 to 7.45).

Similarly, there were no statistically significant differences in MDA levels in septic patients according to the microorganism responsible for sepsis (*P* = 0.79): Gram-positive 2.66 (1.47 to 5.37), Gram-negative 3.21 (1.77 to 4.36), fungii 2.85 (2.22 to 3.90) and anaerobe 2.61 (1.51 to 3.25).

Serum MDA levels were associated with 30-day mortality (hazard ratio = 1.05; 95% confidence interval = 1.02 to 1.09; *P* = 0.005) and 6-month mortality (hazard ratio = 1.05; 95% confidence interval = 1.02 to 1.09; *P* = 0.003) after controlling for lactic acid levels, APACHE-II, diabetes mellitus, bloodstream infection and chronic renal failure (Table 4); thus, each increase of 1 nmol/mL in serum MDA level increased the 30-day and 6-month mortality rate by 5%.

**Table 4:** Cox regression analyses to predict 30-day and 6-month mortality

	**Hazard ratio**	**95% confidence interval**	***P*-value**
**Model: mortality at 30 days**			
MDA serum levels at first day	1.05	1.02 to 1.09	0.005
Lactic acid levels at first day	1.11	1.06 to 1.16	<0.001
APACHE-II	1.03	1.01 to 1.04	0.007
Diabetes mellitus	1.46	0.97 to 2.22	0.07
Bloodstream infection	0.85	0.49 to 1.48	0.56
Chronic renal failure	1.16	0.61 to 2.21	0.66
**Model: mortality at 6 months**			
MDA serum levels at first day	1.05	1.02 to 1.09	0.003
Lactic acid levels at first day	1.11	1.06 to 1.16	<0.001
APACHE-II	1.02	1.01 to 1.04	0.003
Diabetes mellitus	1.55	1.07 to 2.25	0.02
Bloodstream infection	0.73	0.44 to 1.23	0.23
Chronic renal failure	0.85	0.45 to 1.62	0.63

APACHE-II, Acute Physiology and Chronic Health Evaluation-II.

**Figure 4:**
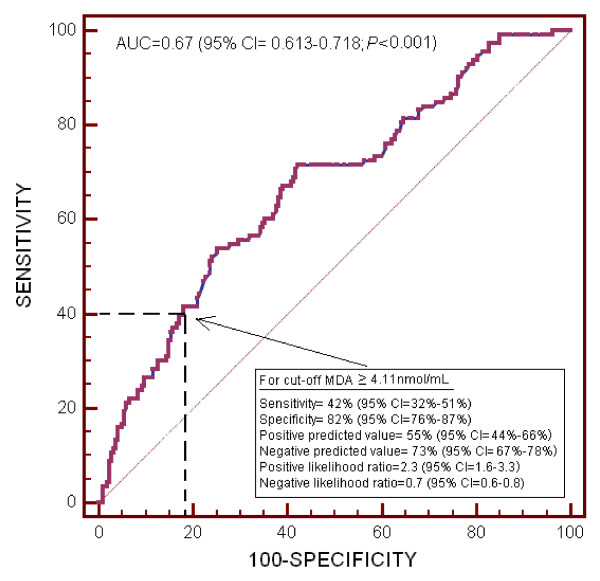
**Receiver operation characteristic analysis using serum MDA levels as a predictor of mortality at 30 days in septic patients.**

We performed a ROC analysis to determine whether serum MDA levels could be used to predict outcomes in septic patients, and found that the area under the curve was 0.67 (95% CI = 0.613 to 0.718; *P* <0.001) (Figure 4).

**Figure 5:**
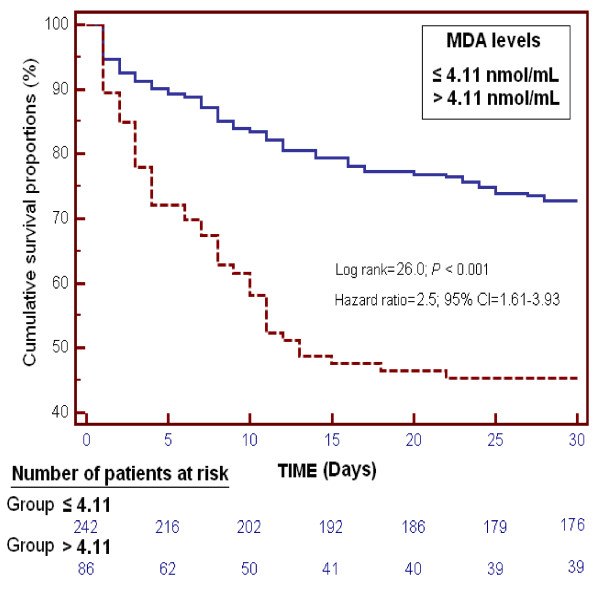
Survival curves at 30 days using serum MDA levels higher or lower than 4.11 nmol/mL.

Kaplan-Meier survival analysis showed that septic patients with serum MDA levels higher than 4.11 nmol/mL had a lower probability of survival at 30 days (log-rank = 26.0; hazard ratio = 2.5 (95% CI = 1.61 to 3.93); *P* <0.001) than patients with lower levels (Figure 5).

**Figure 6:**
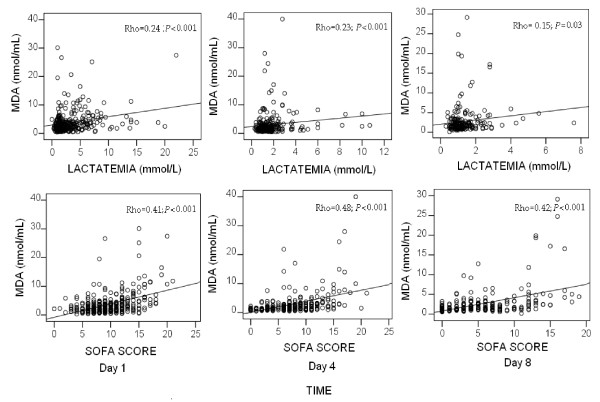
**Correlation of serum MDA levels with lactatemia and SOFA score at days 1, 4 and 8 in severe septic patients.** SOFA, Sepsis-related Organ Failure Assessment score. We used Bonferroni correction to control the multiple testing problem (0.05/6 = 0.008). All *P*-alues lower than 0.008 were considered statistically significant.

We found that serum MDA levels in septic patients positively correlated with lactic acid levels and SOFA score at days 1, 4 and 8 (Figure 6).

## Discussion

To our knowledge, this is largest series providing data on the oxidative state in septic patients. The most relevant new findings were that non-survivors at 30 days showed persistently higher MDA serum levels during the first week than survivors, reflecting a state of lipid hyperoxidation.

We found that severe septic patients had higher serum levels of MDA than healthy controls. These findings are consistent with the results of other small series [6-9] and our previous series [12]. In addition, we found higher MDA serum levels in septic patients at days 4 and 8 of diagnosis than in healthy controls.

In our study, non-surviving septic patients had significantly higher MDA serum levels than survivors at Day 1. These findings are consistent with the results of a small series with 12 septic patients [6] and our previous series [12]. In addition, we found that non-surviving septic patients had significantly higher serum MDA levels than survivors at days 4 and 8.

Lactic acid levels appear to be more strongly associated with 30-day and 6-month mortality than MDA levels. However, MDA levels may be used not only to predict poor outcomes, but also provide insights for a novel therapeutic strategy (that is, antioxidants) that lactate measurement would not provide.

Another aspect of our study is that serum MDA levels could be used as a biomarker to predict the clinical outcome of septic patients according to the results of ROC curve analysis to predict 30-day mortality, although the test properties (sensitivity, specificity, positive and negative predicted values, and positive and negative likelihood ratios) for MDA to predict 30-day mortality are moderate.

In addition, we found that serum MDA levels in survivors decreased significantly during the first week. However, we did not find a significant decrease of serum MDA levels in non-survivors during the first week, although it is possible that the high variability in serum MDA levels and relatively low sample size on days 4 and 8 in non-survivor patients could have influences in this absence of statistically significant differences.

Interestingly, Toufekoula *et al*. have described a compartmentalization of MDA in septic patients [10]. They found higher MDA levels in patients with hepatic dysfunction or acute respiratory distress syndrome (ARDS) compared with patients without organ failures, lower MDA levels in patients with renal dysfunction compared with patients without organ failures, and no differences between patients with cardiovascular dysfunction and without organ failures. However, we found higher MDA levels in septic patients with respiratory, hematologic, hepatic, cardiovascular or renal dysfunction than in control subjects. In a study of septic patients by Ware *et al*. [18], circulating levels of F2-isoprostanes and isofurans as lipid peroxidation products were measured; the authors found that circulating levels of these peroxidation products were associated with renal, hepatic and coagulation failure but not with circulatory or pulmonary failure. In addition, Toufekoula *et al*. found different time-kinetics of serum MDA levels between surviving and non-surviving patients in relation to the type of failing organ, specifically, higher MDA levels in survivors from hepatic dysfunction or from ARDS with cardiovascular dysfunction, and lower MDA levels in survivors from renal dysfunction. However, we found higher MDA levels in non-survivors than in survivors regardless of which organ was affected.

In the study by Andresen *et al*., circulating levels of MDA increased during the follow-up and were higher at 72 hours than at the time of diagnosis [9]. However, we found that MDA levels decreased during follow-up and were higher at Day 1.

We did not find differences in MDA levels according to the microorganism responsible for sepsis, in concordance with the results reported by Toufekoula *et al*. [10]. These findings suggest that oxidative stress is more a function of the general host response to sepsis rather than attributable to a specific microorganism.

Interestingly, we observed a significant correlation between serum MDA levels and several indicators of severity in sepsis, including lactic acid and SOFA score during the first week. Previous studies have reported a positive correlation between MDA and severity in septic patients [9,11]. Andresen *et al*. found a positive correlation between peak MDA and peak lactate levels, and that peak MDA level occurred at 72 hours; however, there was no correlation at Day 1 [9]. In the study by Goode *et al*., patients with three or more failing organs showed higher MDA levels than patients with fewer failing organs [11]. Thus, the new finding of our study was a positive correlation between serum MDA levels and sepsis severity at Day 1 and during the first week.

The discrepancies between our findings and those of Toufekoula *et al*. [10] may be due to lower severity in the patients included in their study where 19% (18/93) of their patients did not have any organ failure, and APACHE-II and SOFA score data were not reported; however, in our study, all patients developed at least one failing organ. In addition, the sample size was lower in the study by Toufekoula *et al*. (93 patients) than in ours (328 patients). The discrepancies between our results and those of Andresen *et al*. [9] may be due to the fact that circulating MDA levels were measured in plasma in their study, not in serum as in ours. In addition, their sample size was also lower (21 patients) than in our study (328 patients).

We found that serum MDA levels were associated with septic mortality, which suggests imbalance in the oxidant state. The imbalance favoring the oxidant state in non-surviving patients could lead to an increase of free radicals and these may contribute to cellular dysfunction, organ failure and finally death [19,20]. The use of melatonin has reduced mortality rate in septic animal models [21-23] and in patients [24-26]. In the study by Fulia *et al*., 20 asphyxiated newborns were randomized to receive melatonin; and patients treated with melatonin showed lower MDA and serum nitrite/nitrate (associated with vasodilatation) levels and lower mortality [24]. In the study by Gitto *et al*., 20 septic newborns were randomized to receive melatonin; those treated with melatonin showed lower serum MDA and 4-hydroxylalkenals (another lipid peroxidation product) levels and lower mortality [25]. In the study by Sahib *et al*., 180 burn patients were randomized to receive different antioxidants (vitamins E and C, zinc sulphate, allopurinol, melatonin and N-acetylcysteine); and the patient groups receiving antioxidants showed higher serum glutathione (that is, a natural antioxidant) levels, and lower serum MDA levels and mortality rate [26]. These findings suggest that the development of modulators of the antioxidant/oxidant state could be used as a new class of drugs for the treatment of severe sepsis; however, more research is necessary to demonstrate these potential benefits. On the other hand, melatonin has been found to play an important role in various functions of the body, including immunoregulation, free radical scavenging, as well as having antioxidant and anti-apoptotic effects [27-31]. It is, therefore, not possible to conclude whether the potential benefits of melatonin are due to its antioxidant effect or due to the combination of all its effects. We found that serum MDA levels were associated with septic mortality; however, it is as yet not possible to affirm that the use of an antioxidant, even if it does lower serum MDA levels, may reduce septic mortality.

The strengths of our study are the large sample size and the follow-up of serum MDA levels throughout the first week after diagnosis. However, some limitations of our study should be recognized. First, the sample size of the study was not pre-determined. Second, the patients were not consecutively included since the study required the collaboration of the research team in each participating hospital and it was not possible to include all septic patients admitted to each ICU; thus, this represents a potential selection bias. Third, we have not reported data on patients lost to follow-up and consent rate. Fourth, the measure of other compounds of oxidant and antioxidant states would be desirable in order to better evaluate this balance. Fifth, the control subjects were recruited from only one center participating in the study, the Hospital Universitario de Canarias (La Laguna, Santa Cruz de Tenerife, Spain); however, they were selected on the basis of non-exposure to antioxidant agents. Sixth, the study lacks some well-established markers of inflammation, such as C-reactive protein or procalcitonin, and it could be interesting to analyze the association between MDA and these biomarkers. Seventh, we did not analyze MDA levels in other critically ill patients to determine whether they could be used as a prognostic biomarker in non-septic critical patients. Eighth, we did not take into account variability in timing of sepsis diagnosis between patients. Ninth, there was a large overlap in serum MDA values between survivors and non-survivors, even though the medians were statistically different; thus, although MDA levels can help to predict 30-day mortality, their clinical utility for an individual patient should be taken with caution. Finally, the association between MDA levels and early septic mortality does not support the notion that antioxidant therapy will decrease mortality.

## Conclusions

To our knowledge, this is the largest series providing data on the oxidative state in septic patients to date. The novel finding is that high serum MDA levels sustained throughout the first week of follow-up were associated with severity and mortality in septic patients.

## Key messages

• Patients with severe sepsis showed higher serum MDA levels than healthy controls.

• Non-surviving septic patients showed higher serum MDA levels than survivors.

• Serum MDA levels could be used to predict outcomes in septic patients.

## Abbreviations

APACHE: Acute physiology and chronic health evaluation; aPTT: Activated partial thromboplastin time; CI: Confidence interval; HR: Hazard ratio; ICU: Intensive Care Unit; INR: International normalized ratio; MDA: malondialdehyde; ROC: Receiver operating characteristic; SOFA: Sepsis-related organ failure assessment score.

## Competing interests

The authors declare that they have no competing interests.

## Authors’ contributions

LLO was responsible for the conception, design and coordination of the study; made substantial contributions to data acquisition, analysis and interpretation; and drafted the manuscript. MMM, ADR, LLA, CD, JSV, JF, JC and JCI made substantial contributions to data acquisition and provided useful suggestions. PAG carried out the determination of serum MDA levels, and made substantial contributions to analysis and interpretation of data. AJ made substantial contributions to analysis and interpretation of data. All authors have read and approved the final manuscript.
